# Integrated Network and Experimental Pharmacology for Deciphering the Medicinal Substances and Multiple Mechanisms of Duhuo Jisheng Decoction in Osteoarthritis Therapy

**DOI:** 10.1155/2020/7275057

**Published:** 2020-11-02

**Authors:** Wenyu Xiao, Weibing Sun, Hui Lian, Juexin Shen

**Affiliations:** Department of Orthopaedics, Shanghai Tenth People's Hospital Chongming Branch, School of Medicine, Tongji University, Shanghai 202157, China

## Abstract

Osteoarthritis (OA) is currently the most common joint disorder worldwide. In last decades, herbal remedies have achieved a significant advancement in the treatment of OA. Duhuo Jisheng Decoction (DHJS), an herbal formula consisting of 15 medicinal herbs, has a long-time practice in OA therapy in China. However, its therapeutic mechanisms have not been comprehensively elucidated. In the present work, integrated network and experimental pharmacology were performed for investigating the therapeutic substances and mechanisms of DHJS. Based on network analysis, the contribution of each herb to OA therapy was evaluated. Furthermore, a series of potential targets and signaling pathways were enriched, which could be involved in the therapeutic effects and mechanisms of DHJS. Further experimental results indicated that DHJS attenuated TNF*α*, IL-6, MMP-1, MMP-9, MMP-13, and ADAMTs-5 expression, inhibited NF-*κ*B and p38 MAPK signaling pathway, activated AMPK-SIRT1 signaling pathway, and suppressed chondrocyte apoptosis, which synergistically contributed to OA therapy. Our work demonstrated that DHJS could be very promising for OA therapy through synergistically acting on multitargets and multipathways.

## 1. Introduction

Osteoarthritis (OA) is currently the most common joint disorder affecting about 250 million people worldwide [1]. With the combined epidemic of ageing and obesity, this syndrome has become a massive health and socioeconomic burden [2]. The pathophysiology of OA is characterized by articular cartilage degradation, subchondral bone remodeling, periarticular muscle weakness, synovial inflammation, and subchondral sclerosis [3]. These pathological changes caused the common symptoms including pain, swelling, and stiffness, which lead to instability and physical disability [4]. Therefore, OA severely impaired the quality of life for affected individuals. The complex pathogenesis of OA involves mechanical, inflammatory, and metabolic factors. For example, excessive production of proinflammatory mediators, including cytokines and chemokines, acts on the synovium to induce inflammation [4]. Catabolic enzymes including matrix metalloproteinases (MMPs), which are released by chondrocytes, are involved in the degradation of extracellular matrix [1]. These factors could tilt the balance between anabolic and catabolic activities of joint tissues toward joint destruction, leading to OA pathogenesis and progression.

Currently, OA management focuses on ameliorating pain, minimizing disability, and enhancing life quality. The therapeutic options recommended in clinic include nonpharmacological and pharmacological interventions. Surgery is offered to those with severe symptoms and for whom conservative approaches have failed [4]. Common pharmacological therapies, such as nonsteroidal anti-inflammatory drugs (NSAIDs), are primarily symptomatic and faced with inefficacy and long-term adverse events [5, 6]. Intra-articular corticosteroids are recommended in case the OA patients have not responded to oral or topical analgesics [7], but the evidence for clinically important benefits of corticosteroids still remains unclear [8]. In addition, the efficacy of glucosamine and chondroitin sulfate in cartilage repair is also controversial [9]. In summary, the limited efficacy and safety issues surrounding the existing medications necessitate other safe and effective treatments.

Herbal remedy, as an important branch of complementary and alternative medicine, has a long tradition in the treatment of OA. Duhuo Jisheng Decoction (DHJS), a traditional Chinese herbal recipe, has been developed and prescribed to treat OA in China for a long time [10]. This formula consists of 15 species of medicinal herbs: *Radix Angelicae pubescentis* (Duhuo), *Herba Taxilli* (Sangjisheng), *Radix Gentianae Macrophyllae* (Qinjiao), *Radix Saposhnikoviae* (Fangfeng), *Herba Asari* (Xixin), *Cortex Cinnamomi* (Rougui), *Poria Cocos* (Fuling), *Rhizoma Chuanxiong* (Chuanxiong), *Radix Paeoniae Alba* (Baishao), *Cortex Eucommiae Ulmoidis* (Duzhong), *Codonopsis Radix* (Dangshen), *Radix Glycyrrhizae* (Gancao), *Radix Angelicae Sinensis* (Danggui), *Radix Achyranthis Bidentatae* (Niuxi), and *Radix Rehmanniae Preparata* (Shudihuang). According to traditional Chinese medicine theory, *Radix Angelicae pubescentis* is monarch drug responding to dispelling wind and eliminating dampness; *Radix Gentianae Macrophyllae*, *Herba Asari*, and *Cortex Cinnamomi* are minister drugs responding to expelling wind, alleviating pain, and eliminating stagnation; *Herba Taxilli*, *Rhizoma Chuanxiong*, *Radix Achyranthis Bidentatae*, and *Cortex Eucommiae Ulmoidis* are assistant drugs responding to nourishing liver and kidney, strengthening the bone, and activating blood circulation; *Radix Glycyrrhizae* is guide drug responding to harmonizing other herbs. Therefore, DHJS can be mainly used to treat arthralgia syndrome, with the effects of eliminating stagnation, removing blood stasis, and also nourishing the liver and kidney [11].

In recent years, several clinical trials showed that DHJS could relieve OA-related symptoms [10, 12], and a latest meta-analysis paper demonstrated that DHJS combined with Western medicine or sodium hyaluronate injection appears to have beneficial outcomes for OA [13]. Based on modern biological techniques, research on the mechanisms of DHJS suggested that this decoction could alleviate OA through promoting chondrocyte proliferation and suppressing chondrocyte senescence and apoptosis [14–16]. However, DHJS is a multiherb formula containing plentiful bioactive ingredients, and its pharmacological efficacy could be derived from the synergistic actions of the multi-ingredients modulating multipathways in a whole system level. Although several studies have provided some clue to the pharmacological mechanism of DHJS, the comprehensive and precise mechanisms of DHJS on OA therapy have not been elucidated. Network pharmacology is an emerged cutting-edge methodology for revealing the synergism law of multicomponent drugs and seeking high efficacy and low toxicity of multiple-target medications [17]. In the present work, integrated network and experimental pharmacology were employed to pursue the scientific acknowledge regarding the principle of formula combination and elucidate the systematic and comprehensive mechanisms of DHJS on OA therapy.

## 2. Materials and Methods

### 2.1. Network Pharmacology-Based Analysis

#### 2.1.1. Construction of DHJS Ingredient Library

The ingredients of each herb in DHJS formula were collected from TCMSP database. TCMSP database provides detailed information of various active ingredients related to traditional Chinese medicines (TCMs), including molecular weight, oral bioavailability, drug likeness, aqueous solubility, and drug targets [18]. In order to gather all reported ingredients in herbal formula, we also performed an extensive literature search for each herb.

#### 2.1.2. Screening Strategy for Active Ingredients in DHJS

Oral bioavailability (OB) is one of the most important pharmacokinetic parameters for an oral administered drug. It is commonly used in drug screening cascades for screening candidate compounds [19]. Drug-likeness (DL) is a qualitative parameter representing how the pharmacokinetic and pharmaceutical properties of a compound correspond in the majority of available drugs [20]. In present work, OB ≥ 25% and DL ≥ 0.18 were set as a threshold for screening candidate ingredients from DHJS ingredient library. We set both of these variables because only those ingredients with OB and DL above the thresholds are likely the potential bioactive compounds contributing to the therapeutic effects [21, 22].

#### 2.1.3. Collection of DHJS Putative Targets and OA Targets

Herbal formula with numerous bioactive ingredients could regulate multiple targets, so target identification is helpful to elucidate the therapeutic mechanism of DHJS. In this study, two specialized databases (TCMSP and STITCH) were used to search the putative targets of the candidate ingredients. Furthermore, considering that the databases are hard to realize real-time update and incorporate all latest studies, we also performed a large-scale text mining of PubMed using the each herbal ingredient as search term and manually extracted relevant targets from literatures ranging from the years 2015 to 2020. The official symbols of DHJS targets were generated through UniProtKB database (https://www.uniprot.org/) with the species limited to “*Homo sapiens*.” Finally, comprehensive ingredient-target interactome of DHJS was constructed in silico. The targets of OA were exported from the human gene database GeneCards (http://www.genecards.org/). The items “Symbol” and “Score” of each gene related to OA were reserved.

#### 2.1.4. Pattern Recognition for Integrated Analysis of DHJS-OA Targets

For all DHJS targets, the repetition number of each target was counted as *n*, and then the same targets were merged. The values of *n* were normalized with the maximum *n* normalized to 1. For OA targets, the “Score” values of genes were normalized through being divided by the maximum value. After normalization, the target scores ranged from 0 to 1, and the target with normalized score 1 was the most relevant target to OA. The number of interaction targets of DHJS and OA was generated with Venn diagram. The overlapped targets with corresponding values of herb targets and OA targets were extracted. Finally, the dataset containing herb targets and OA targets with corresponding values were constructed. After dataset construction, pattern recognition models including principal components analysis (PCA) and hierarchical cluster analysis (HCA) were used to resolve the relevance between herb targets and OA targets. The dataset was exported to the SIMCA-P software (version 12.0) and Heml (version 1.0.3) to perform PCA and HCA, respectively.

#### 2.1.5. Network Construction and Analysis

To explore the multiscale mechanisms of therapeutic action of DHJS in OA, an integrated network consisting of “herb-ingredient network”, “ingredient-target network,” and “disease-target network” was constructed by Cytoscape 3.2.0. In the graphic network, herbal formula, ingredients, targets, and disease were described by nodes, and the intermolecular interactions were encoded by edges. The coverage scale of the targets of each herb and the contribution degree of the targets of each herb were applied to the analysis of the obtained network.

#### 2.1.6. Protein-Protein Interaction Construction and Analysis

The DHJS-OA targets were imported into STRING web server (http://www.string-db.org/) for predicting protein-protein interaction (PPI). In PPI, the minimum required interaction score was set as 0.9 and max number of interactors was set as 5. Subsequently, gene ontology (GO) enrichment analysis was performed to extract the functional annotations of these targets.

#### 2.1.7. Enrichment Analysis

Kyoto Encyclopedia of Genes and Genomes (KEGG) pathway enrichment analysis was performed to extract the canonical pathways and highly associated proteins. *p* values were given in enrichment analysis, and smaller *p* values suggested greater enrichment. To further characterize the molecular mechanism of DHJS on OA, the target-pathway network was generated using Cytoscape 3.2.0 based on the pathway data.

### 2.2. Experimental Validation

#### 2.2.1. Preparation of DHJS Extract

The herbs in DHJS were purchased from Tongrentang (Shanghai, China). Then these herbs were identified by experts from School of Chinese Materia Medica, Shanghai University of Chinese Medicine (Shanghai, China). Herbs were mixed and extracted with the standard methods according to Chinese Pharmacopoeia 2015. Briefly, herbs were soaked in distilled water and boiled for 30 min twice and then the solution was filtered and concentrated with a rotary evaporator. The product was dissolved in DMSO to obtain a 1 g/mL stock solution. The obtained solution was filtered twice (0.22 *μ*m) and the filtrate was stored at 4°C.

#### 2.2.2. Cell Isolation and Culture

All animal procedures were approved by the Institutional Animal Care and Use Committee of Shanghai Tenth People's Hospital Chongming Branch. Male Sprague-Dawley rats (8 weeks old, 200 ± 20 g) were purchased from Super-BK Laboratory Animal Co. (Shanghai, China) and maintained under specific pathogen-free (SPF) conditions. Chondrocytes were isolated from rat articular cartilage and cultured as previously described [23]. The second-passage (P2) chondrocytes cultured until ∼80% confluency were used in the study.

#### 2.2.3. Cell Viability Assay

Briefly, chondrocytes were seeded in 96-well plates and then incubated with different concentrations of DHJS (20, 50, 100, 200, 500, and 1000 *μ*g/mL) in the presence or absence of recombinant rat IL-1*β* (10 ng/mL) (Beyotime Institute of Biotechnology, Shanghai, China) for 24, 48, or 72 h. Subsequently, MTT assay was conducted to assess the effect of DHJS on cell viability.

#### 2.2.4. Real-Time PCR

Chondrocytes were treated with different concentrations of DHJS (50, 100, and 200 *μ*g/mL) for 2 h, followed by stimulation with or without IL-1*β* (10 ng/mL) for 24 h. Total RNA was extracted using Trizol reagent (Takara, Shiga, Japan) according to instructions and was reverse-transcribed to cDNA using a PrimeScript RT reagent kit (Takara, Shiga, Japan). Subsequently, real-time PCR was performed using SYBR® GreenER SuperMix (Takara, Shiga, Japan) and the results were analyzed using the 2^−(△△Ct)^ method. The primers used were as follows: *β*-actin: forward 5′-GGAGATTACTGCCCTGGCTCCTA-3′, reverse 5′-GACTCATCGTACTCCTGCTTGCTG-3′; IL-6: forward 5′-ATTGTATGAACAGCGATGATGCAC-3′, reverse 5′-CCAGGTAGAAACGGAACTCCAGA-3′; TNF*α*: forward 5′-TCAGTTCCATGGCCCAGAC-3′, reverse 5′-GTTGTCTTTGAGATCCATGCCATT-3′; MMP-1: forward 5′-CTGAAGGTGATGAAGCAGCC-3′, reverse 5′-AGTCCAAGAGAATGGCCGAG-3′; MMP-13: forward 5′-TGATGATGAAACCTGGACAAGCA-3′, reverse 5′-GAACGTCATCATCTGGGAGCA-3′; ADAMTs-5: forward 5′-AGAGTCCGAACGAGTTTACG-3′, reverse 5′-GTGCCAGTTCTGTGCGTC-3′.

#### 2.2.5. Western Blot

Proteins were extracted from cells using RIPA buffer and the protein concentration was determined using BCA protein assay kit (Thermo Scientific, IL, USA). Equal amounts of protein were separated by 10% SDS-PAGE and transferred onto PVDF membranes (Millipore, MA, USA). After blocking, PVDF membranes were incubated with primary antibodies against p65, p-p65, p38, p-p38, SIRT1, Bax, Bcl2, c-caspase3, and *β*-actin (Abcam Inc., MA, UK), followed by incubation with HRP-conjugated secondary antibody (Cell Signaling Technology Inc., MA, USA). The protein bands were visualized with Ultra Signal chemiluminescence reagents (4A Biotech Co., Ltd., Beijing, China).

#### 2.2.6. Statistical Analysis

Data was shown as mean ± standard deviation (SD). Statistical differences of data were evaluated using the independent-samples *T*-test analysis or one-way analysis of variance using SPSS 16.0 software. *p* value <0.05 was considered statistically significant.

## 3. Results

### 3.1. Network Pharmacology-Based Analysis

#### 3.1.1. Candidate Bioactive Ingredients in DHJS

A large number of ingredients in DHJS were obtained from TCMSP database (Table 1). Their corresponding properties were also collected to construct DHJS ingredient library. Subsequently, collected ingredients were subjected to OB and DL screening. As a result, total 77 ingredients were included and regarded as candidate compounds. The detailed information of 77 screened ingredients is shown in Table 1.

#### 3.1.2. Collection of DHJS Putative Targets and OA Targets

Based on screening in TCMSP and STITCH databases, as well as supplemented text mining of PubMed, 77 candidate compounds yielded 359 targets after deleting duplicates. Total 2854 disease-related targets were extracted from GeneCards database. Finally, the library of DHJS targets and OA targets was constructed based on the procedures described in the above method.

#### 3.1.3. Pattern Recognition for Integrated Analysis of DHJS-OA Targets

**Figure 1:**
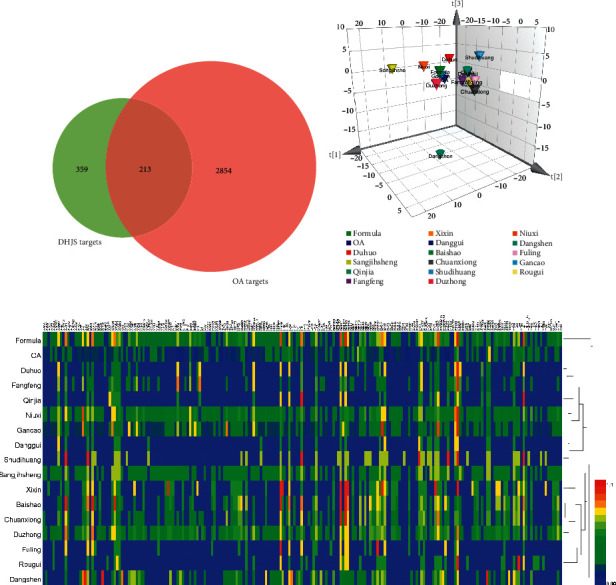
Integrated analysis of DHJS-OA targets. Venn diagram of DHJS and OA targets (a). Scores scatter 3D plot from PCA analysis of DHJS-OA target dataset (b). Heatmap from HCA analysis of DHJS-OA target dataset (c).

Total 359 DHJS targets were mapped with 2854 OA targets to generate 213 interaction targets (Figure 1(a)). Subsequently, the dataset containing DHJS targets with values and OA targets with values was imported into SIMCA and Heml software for pattern identification analysis. In SIMCA, PCA was used to obtain discrimination by predicting group membership. Scores scatter 3D plot from PCA showed that there was no clear separation between DHJS and OA, suggesting the high integration degree of DHJS targets and OA targets (Figure 1(b)). In addition, Dangshen, Sangjisheng, and Shudihuang showed obvious separation from other herbs, DHJS formula, and OA. Considering the distance of OA group to herb groups, Duhuo, Gancao, Niuxi, Duzhong, and Fangfeng were among the nearest groups to OA group. In Heml, heatmap from HCA analysis showed similar result to that from PCA analysis (Figure 1(c)). As expected, DHJS group and OA group were assigned into a near cluster since they shared similar target profile. Moreover, Dangshen was the farthest group from OA group, suggesting the obvious difference between their targets.

#### 3.1.4. Network Construction and Analysis

**Figure 2:**
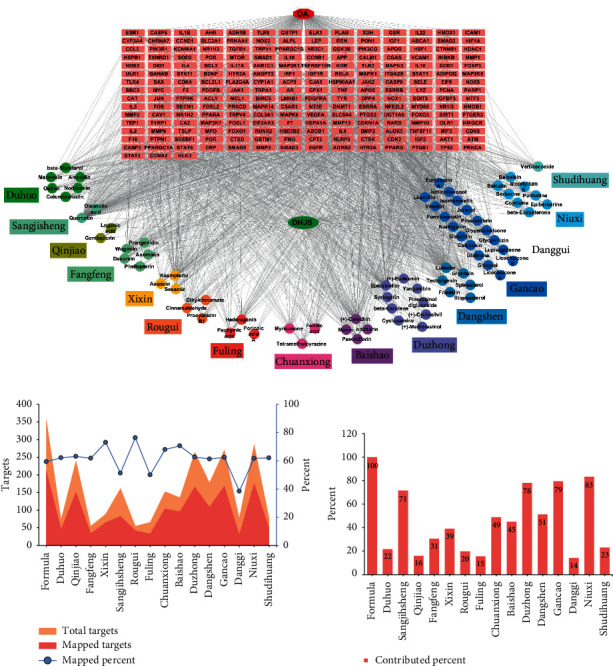
Network construction and analysis. Network diagram constructed by linking ingredient with putative targets (a). The nodes represent candidate compounds in DHJS which are shown as colorful circle, and the targets are indicated by light red fusiform. The total targets and DHJS-OA mapped targets generated by each herb (b). The contribution of each herb in DHJS formula to the interaction targets (c).

77 ingredients and 213 interaction targets were imported into Cytoscape software for network construction. The network linking the candidate ingredients with their putative targets was plotted (Figure 2(a)). The affiliation of ingredients with herbs was also described. The ingredients with coexistence in several herbs were listed in only one herb based on the anticlockwise sort in network plot. As a result, the generated network diagram consisted of 288 nodes and 1985 edges. This diagram showed a complex interactive relationship between “one compound-multiple targets” and “one target-multiple compounds.” Additionally, there was no ingredient having affiliation with Danggui because the ingredients found in Danggui were also found in other herbs. Furthermore, all targets linked with verbascoside contained in Shudihuang were also linked with other ingredients in other herbs.

As depicted in Figure 2(b), the herb with more ingredients generated more targets and presented more probability to cover OA targets. Danggui contained the smallest number of ingredients and thus generated the least targets. The median of mapped percent of herbs was about 60%, and the herbs Danggui, Fuling, and Xixin were the top three at counting backward. Considering the contribution of each herb to DHJS-OA mapped targets, the herbs Niuxi, Gancao, and Duzhong were the top three, while the herbs Danggui, Fuling, and Qinjiao were the top three at counting backward (Figure 2(c)).

#### 3.1.5. Protein-Protein Interaction Construction and Analysis

**Figure 3:**
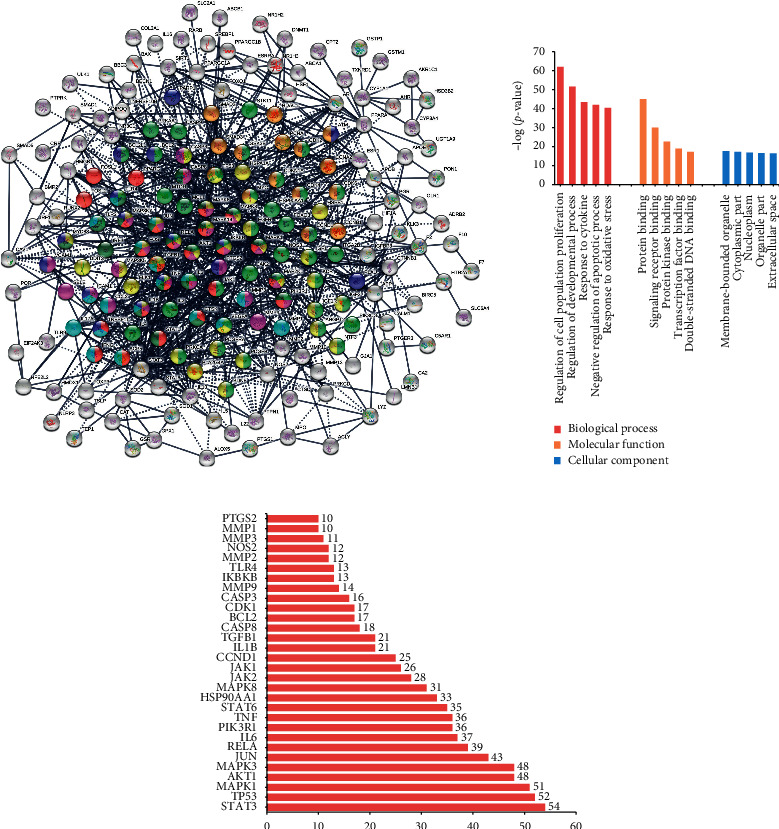
PPI construction and analysis. The PPI network generated on DHJS-OA mapped targets (a). The top 5 significantly enriched BP, CC, and MF categories based on gene ontology (b). The top 30 candidate targets extracted from PPI according to the node degree (c).

The PPI network was constructed in STRING as shown in Figure 3(a). The number of nodes was 213 and the number of edges was 1154. The nodes strongly linked with OA-related pathways were labeled with color. The GO information including biological process (BP), cellular component (CC), and molecular function (MF) terms was obtained. The top 5 significantly enriched terms in BP, CC, and MF categories are shown in Figure 3(b). The results indicated that DHJS could exert biological actions via protein binding, kinase binding, and transcription factor binding in organelle, cytosol, nucleoplasm, and extracellular space to exert its therapeutic effects.  Figure 3(c) depicts the top 30 candidate targets according to the node degree which indicated the degree of importance to OA therapy. These targets were believed as crucial proteins involved in OA pathogenesis and pharmacologic therapeutic.

#### 3.1.6. Pathway Enrichment Analysis

**Figure 4:**
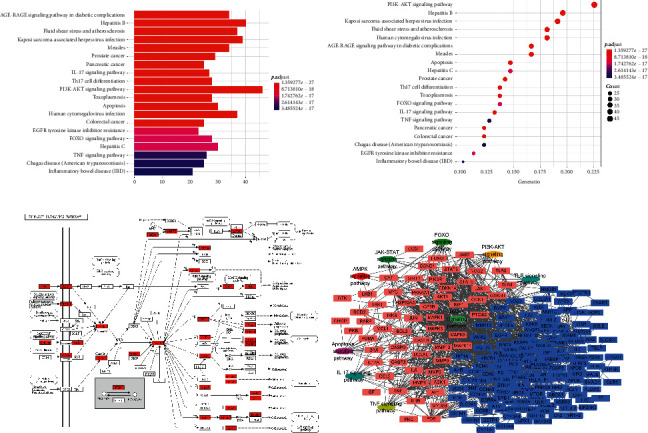
Pathway enrichment analysis. The top 20 pathways extracted based on KEGG enrichment analysis (a). The enriched PI3K-AKT pathway with mapped targets labeled red (b). The target-pathway network for DHJS on OA, in which the red nodes represent the most potential targets and the oval nodes represent the most potential pathways (c).

To further characterize the molecular mechanism of DHJS on OA treatment, we carried out pathway-based functional enrichment analysis. Total 70 KEGG pathways were systematically enriched and extracted. Among them, top 20 KEGG pathways are shown in Figure 4(a). Total 8 signaling pathways, including PI3K-AKT signaling pathway, FOXO signaling pathway, TNF signaling pathway, JAK-STAT signaling pathway, MAPK signaling pathway, AMPK-SIRT1 signaling pathway, IL-17 signaling pathway, TLR signaling pathway, and apoptosis signaling pathway, were assigned to be the positive pathways associated with OA. A representative enriched KEGG pathway, PI3K-AKT signaling pathway, is shown in Figure 4(b). Based on the extraction of labeled targets in these pathways, a target-pathway network was generated by linking extracted targets with their corresponding significant signaling pathways (Figure 4(c)). This network illustrated the most potential targets and pathways which played a pivotal role in the therapeutic efficacy of DHJS formula for OA therapy.

### 3.2. Experimental Validation

#### 3.2.1. Effect of DHJS on Chondrocyte Viability

**Figure 5:**
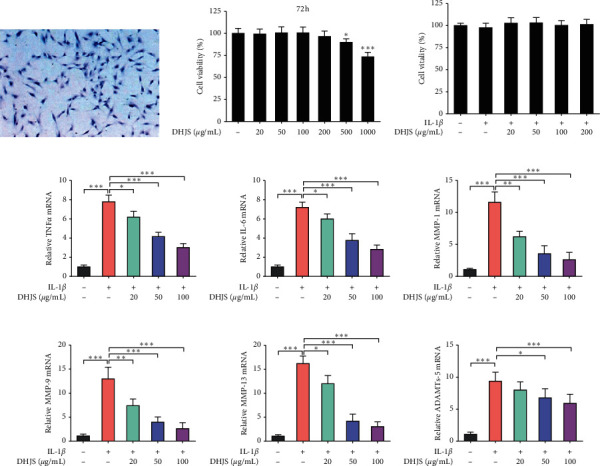
Effects of DHJS on cell viability and IL-1*β*-induced expression of TNF*α*, IL-6, MMPs, and ADAMTs-5 in rat chondrocytes. Identification of rat primary chondrocytes (a). Rat chondrocytes were shaped like spindles with a protuberance, in which proteoglycans were stained purple by toluidine blue. Effects of DHJS on cell viability of chondrocytes (b). Effects of DHJS on cell viability of IL-1*β*-induced chondrocytes (c). Effects of DHJS on IL-1*β*-induced expression of TNF*α* (d), IL-6 (e), MMP-1 (f), MMP-3 (g), MMP-9 (h), and ADAMTs-5 (i) in rat chondrocytes. Data are expressed as mean ± SD (*n* = 3). ^*∗*^*p* < 0.05, ^*∗∗*^*p* < 0.01, and ^*∗∗∗*^*p* < 0.001, compared between the marked groups.

Isolated primary chondrocytes were identified by toluidine blue staining. The primary chondrocytes were shaped like spindles with a protuberance, and proteoglycans in rat primary chondrocytes were stained purple by toluidine blue (Figure 5(a)). DHJS showed no significant cytotoxicity at doses ranging from 20 to 200 *μ*g/mL, while higher dose of DHJS (≥500 *μ*g/mL) significantly inhibited the cell viability (Figure 5(b)). Likewise, DHJS (≤200 *μ*g/mL) exerted no significant effect on the viability of IL-1*β*-induced chondrocytes (Figure 5(c)).

#### 3.2.2. DHJS Suppresses IL-1*β*-Induced TNF*α*, IL-6, MMPs, and ADAMTs-5 Expression in Chondrocytes

In OA, large amounts of TNF*α*, IL-6, MMPs, and ADAMTs-5 are released with the occurrence of inflammation and resulted in ECM dissolution. In the present work, the effects of DHJS on the mRNA expression of these factors were examined in chondrocytes. As expected, IL-1*β* treatment induced significant overexpression of TNF*α*, IL-6, MMP-1, MMP-9, MMP-13, and ADAMTs-5 genes in primary chondrocytes (Figures 5(d)–5(i)). Interestingly, DHJS pretreatment significantly alleviated the overexpression of TNF*α*, IL-6, MMP-1, MMP-9, MMP-13, and ADAMTs-5 genes in IL-1*β*-induced chondrocytes (Figures 5(d)–5(i)).

#### 3.2.3. Effect of DHJS on Dominating Signaling Pathways in IL-1*β*-Induced Chondrocytes

**Figure 6:**
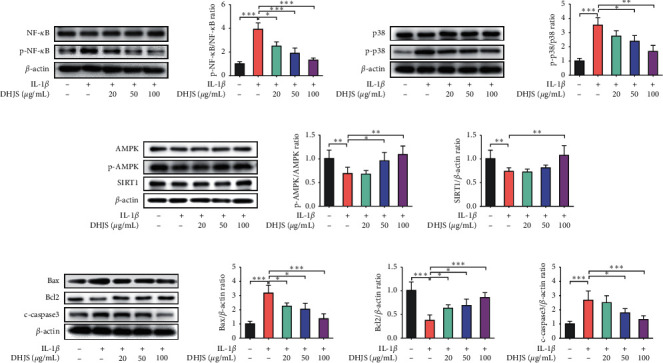
Effects of DHJS on protein expression of NF-*κ*B, p38, AMPK, SIRT1, Bax, Bcl2, and c-caspase3 in IL-1*β*-induced chondrocytes. DHJS reduced IL-1*β*-induced NF-*κ*B phosphorylation (a). DHJS reduced IL-1*β*-induced p38 phosphorylation (b). DHJS increased AMPK phosphorylation and SIRT1 expression in IL-1*β*-induced chondrocytes (c). DHJS reduced Bax and c-caspase3 expression and increased Bcl2 expression in IL-1*β*-induced chondrocytes (d). Data are expressed as mean ± SD (*n* = 3). ^*∗*^*p* < 0.05, ^*∗∗*^*p* < 0.01, and ^*∗∗∗*^*p* < 0.001, compared between the marked groups.

To obtain insight into the mechanisms of DHJS on OA therapy, the expression levels of several key proteins involved in dominating signaling pathways were detected in IL-1*β*-induced chondrocytes with or without DHJS pretreatment (Figures 6(a)–6(d)). As shown in Figure 6(a), IL-1*β* inducement significantly increased the phosphorylation of NF-*κ*B in comparison with control group (*p* < 0.001), while DHJS pretreatment significantly inhibited the IL-1*β*-induced phosphorylation of NF-*κ*B (*p* < 0.05). Likewise, significant p38 phosphorylation was found in IL-1*β*-induced chondrocytes (*p* < 0.001), which was effectively alleviated by DHJS pretreatment (Figure 6(b)). In addition, IL-1*β* inhibited the AMPK-SIRT1 pathway by suppressing AMPK phosphorylation and SIRT1 protein expression, which was also restored by DHJS pretreatment (Figure 6(c)). Furthermore, several apoptosis-related markers were evaluated. The results indicated that IL-1*β* induced significant upregulation of proapoptotic c-caspase3 and Bax proteins and downregulation of antiapoptotic Bcl2 protein (Figure 6(d)). Fortunately, DHJS pretreatment abrogated IL-1*β*-induced dysregulation of apoptosis-related proteins (Figure 6(d)). Taken together, the above results indicate that DHJS could suppress NF-*κ*B and p38 activation, activate AMPK-SIRT1 pathway, and inhibit apoptosis pathway.

## 4. Discussion

The pathophysiology of OA is still evolving, from being viewed as a cartilage-limited disease to a multifactorial disease [4]. It has been demonstrated that a wide range of underlying pathways lead to similar outcomes of joint destruction in OA [24]. The proposed mechanistic pathways include increased inflammatory components [25], mechanical overload [26], metabolic alterations [27], and cell senescence [28]. For detail, factors involved in inflammatory state include IL-1*β*, IL-6, TNF*α*, PGE2, NO, and ROS; factors involved in matrix degradation include MMP-1, MMP-9, MMP-13, and ADAMTs-5; factors involved in osteoclast differentiation include receptor activator of nuclear factor-*κ*B ligand (TNFSF11) and sclerostin; factors involved in chondrocyte senescence include cytokines, chemokines, ROS, and HIF-1*α*. Therefore, these factors play crucial roles in OA pathogenesis and also represent the potential targets to treat OA.

In last decades, herbal remedies have seen significant advancement against OA [29, 30]. DHJS formula consisting of 15 medicinal herbs has a clinical practice in OA therapy for a long time [11–13]. It was believed that the synergistic actions of bioactive ingredients respond to therapeutic efficacy of this formula for OA [14–16]. However, our study indicated that the ingredients contained in each herb and their corresponding targets showed some overlap. For instance, the candidate ingredients found in Danggui were also found in other herbs. The putative targets generated by the only candidate ingredient in Shudihuang were also found in other herbs. Furthermore, the herbs Danggui and Shudihuang showed much little contribution to DHJS-OA mapped targets. Therefore, these results suggested that the ingredients and targets of Danggui and Shudihuang could be supplemented by other herbs which could be modified in DHJS formula for OA therapy. In addition, the contribution of each herb to OA therapy was different. PCA and HCA analysis indicated that Duhuo, Gancao, Niuxi, Duzhong, and Fangfeng were highly associated with OA therapy. It was consistent with that these herbs contributed the most DHJS-OA targets. Indeed, these herbs have also been widely reported to exert protective effects on joint cartilages in OA therapy [31–35]. However, the herbs Qinjiao and Fuling also showed much little contribution to DHJS-OA mapped targets, and there are few related reports about the using of Qinjiao and Fuling for OA therapy. These results suggested that the evidence of applying Qinjiao and Fuling for OA treatment was insufficient. In summary, in DHJS formula, the herbs including Duhuo, Gancao, Niuxi, Duzhong, and Fangfeng could exert greater contribution to OA therapy while herbs including Danggui, Shudihuang, Qinjiao, and Fuling might exert less contribution to OA therapy. However, further comparative pharmacological studies need to be carried out to validate such conclusion.

Although clinical trials demonstrated that DHJS could relieve OA-related symptoms [10, 12, 13] and a few reports indicated the relevant therapeutic mechanisms [14–16], the comprehensive and precise mechanisms of DHJS on OA therapy have not been elucidated. In the present work, a series of potential targets and interactive pathways were proposed based on enhanced network pharmacology, which were believed to play a pivotal role in the therapeutic efficacy of DHJS formula for OA therapy. IL-1*β* is a key inflammation cytokine in OA pathogenesis and progression. IL-1*β* stimulates the production of several inflammatory mediators, such as iNOS, TNF*α*, and IL-6, which contribute to chondrocytic dysfunction [36, 37]. Moreover, IL-1*β* also promotes the secretion of MMPs such as MMP-1, MMP-13, and ADAMTSs-5 in chondrocytes, which cause cartilage degeneration and ECM destruction [38, 39]. Based on network pharmacology, a series of key targets were enriched from network analysis, such as PTGS2, MMP-1, MMP-9, MMP-13, iNOS, IL-1*β*, TNF*α*, and IL-6. These results suggested that DHJS could regulate these targets to exert beneficial effects on OA. As expected, DHJS apparently inhibited IL-1*β*-induced TNF*α*, IL-6, MMP-1, MMP-9, MMP-13, and ADAMTs-5 expression in rat chondrocytes, which were basically consistent with the results of network analysis.

Next, we validated the therapeutic mechanisms of DHJS on OA. Several dominating signaling pathways were extracted from KEGG enrichment. Based on pathway enrichment, DHJS could regulate JAK-STAT signaling pathway for modulating G1/S phase transition to promote chondrocyte proliferation, which was consistent with a previous report [15]. However, DHJS at doses ranging from 20 to 200 *μ*g/mL showed no significant effect on chondrocyte viability, suggesting that DHJS might be hard to promote chondrocyte proliferation. NF-*κ*B pathway is profoundly involved in the regulation of inflammatory mediators in OA progression [40]. Upon stimulation, the activated NF-*κ*B molecules trigger the expression of proinflammatory factors to induce the destruction of articular joint [40]. Therefore, inhibition of NF-*κ*B pathway could represent a promising therapeutic option for OA treatment. Pathway enrichment indicated that DHJS could target NF-*κ*B pathway, and in vitro experiment confirmed this conjecture in which DHJS significantly inhibited NF-*κ*B phosphorylation. Moreover, emerging evidence has demonstrated that p38 MAPK signal pathway was involved in OA development, and inhibiting the activation of p38 MAPK pathway may be a new target for OA treatment [41]. In the present work, the phosphorylation of p38 in IL-1*β*-stimulated chondrocytes was significantly reduced by DHJS, suggesting that inhibiting p38 MAPK pathway represented one of the mechanisms of DHJS to treat OA. In addition, pathway enrichment indicated that DHJS could regulate AMPK-SIRT1 pathway to enhance oxidative defense, and cell experiment verified that DHJS activated AMPK-SIRT1 pathway in IL-1*β*-induced chondrocytes. Moreover, Liu et al. reported that DHJS suppressed cell apoptosis to alleviate intervertebral disc degeneration [16]. Consistently, our study suggested DHJS could mediate apoptosis signaling pathway to protect chondrocyte against apoptosis, which was also confirmed by in vitro experiment. In summary, DHJS could have great potential in OA therapy through synergistic actions derived from acting on these multitargets and multipathways.

In conclusion, integrated network and experimental pharmacology were employed, which provided a comprehensive investigation to understand the medicinal substances and molecular mechanism of DHJS for OA treatment. The results demonstrated that DHJS could attenuate OA through decreasing TNF*α*, IL-6, MMP-1, MMP-9, MMP-13, and ADAMTs-5 levels, and the corresponding mechanisms could involve inhibiting NF-*κ*B and p38 MAPK signaling pathways, activating AMPK-SIRT1 signaling pathway, and reversing chondrocyte apoptosis. Due to the multifactorial pathogenesis of OA, DHJS with synergistic effects on multitargets and multipathways could have considerable potential in OA therapy.

## Figures and Tables

**Table 1:** Information for the ingredients of each herb in DHJS.

No.	Herb	Number of ingredients			Name of ingredients
		Total	OB & DL screening	Target screening	
1	Duhuo	99	13	6	Ammidin, beta-sitosterol, columbianadin, marmesin, nodakenin, and osthol
2	Sangjisheng	46	4	3	Beta-sitosterol, oleanolic acid, and quercetin
3	Qinjiao	27		4	Beta-sitosterol, gentiopicrin, loganic acid, and oleanolic acid
4	Fangfeng	173	26	8	Ammidin, anomalin, beta-sitosterol, decursin, marmesin, phellopterin, prangenidin, and wogonin
5	Xixin	192	10	3	Asarinin, kaempferol, and sesamin
6	Rougui	100	3	3	Cinnamaldehyde, ethyl cinnamate, and procyanidin B1
7	Fuling	34	19	3	Hederagenin, pachymic acid, and poricoic acid A
8	Chuanxiong	189	12	4	Beta-sitosterol, ferulic acid, myricanone, and tetramethylpyrazine
9	Baishao	85	15	7	Albiflorin, beta-sitosterol, (+)-catechin, kaempferol, mairin, oleanolic acid, and paeoniflorin
10	Duzhong	147	39	13	Beta-carotene, beta-sitosterol, (+)-cycloolivil, cyclopamine, epicatechin, (+)-Eudesmin, kaempferol, mairin, (+)-medioresinol, pinoresinol diglucoside, quercetin, syringetin, and yangambin
11	Dangshen	134	26	6	Friedelin, glycitein, spinasterol, stigmasterol, tectorigenin, and luteolin
12	Gancao	280	101	28	Beta-sitosterol, calycosin, eurycarpin A, formononetin, glabridin, glabrone, glycyrol, glycyrrhizin, glypallichalcone, isolicoflavonol, isorhamnetin, jaranol, kaempferol, licochalcone A, licochalcone B, liquiritin, lupiwighteone, naringenin, oleanolic acid, pinocembrin, quercetin, and vestitol
13	Danggui	125	3	3	Beta-sitosterol, ferulic acid, and stigmasterol
14	Niuxi	176	22	15	Baicalin, baicalein, berberine, beta-ecdysterone, beta-sitosterol, coptisine, epiberberine, inophyllum E, kaempferol, oleanolic acid, palmatine, quercetin, spinasterol, stigmasterol, and wogonin
15	Shudihuang	76	6	3	Beta-sitosterol, stigmasterol, and verbascoside

## Data Availability

The analyzed data sets generated during the present study are available from the corresponding author on reasonable request.
